# The mechanical properties of tibiofemoral and patellofemoral articular cartilage in compression depend on anatomical regions

**DOI:** 10.1038/s41598-021-85716-2

**Published:** 2021-03-17

**Authors:** Heng Li, Jinming Li, Shengbo Yu, Chengwei Wu, Wei Zhang

**Affiliations:** 1 State Key Laboratory of Structure Analysis for Industrial Equipment, Department of Engineering Mechanics, Dalian University of Technology, Dalian, 116024 China; 2 Department of Anatomy, College of Basic Medical Sciences, Dalian Medical University, Dalian, 116044 China

**Keywords:** Materials science, Biological physics

## Abstract

Articular cartilage in knee joint can be anatomically divided into different regions: medial and lateral condyles of femur; patellar groove of femur; medial and lateral plateaus of tibia covered or uncovered by meniscus. The stress–strain curves of cartilage in uniaxially unconfined compression demonstrate strain rate dependency and exhibit distinct topographical variation among these seven regions. The femoral cartilage is stiffer than the tibial cartilage, and the cartilage in femoral groove is stiffest in the knee joint. Compared with the uncovered area, the area covered with meniscus shows the stiffer properties. To investigate the origin of differences in macroscopic mechanical properties, histological analysis of cartilage in seven regions are conducted. The differences are discussed in terms of the cartilage structure, composition content and distribution. Furthermore, the commonly used constitutive models for biological tissues, namely Fung, Ogden and Gent models, are employed to fit the experimental data, and Fung and Ogden models are found to be qualified in representing the stiffening effect of strain rate.

## Introduction

Cartilage is an essential part of the tibiofemoral and patellofemoral joints and provides a shock-resistant, cushioning, and friction-reducing surface for the two joints^1^. According to functional differences and anatomy definition, the cartilage of the two joints can be classified into seven regions: medial condyle of femur (FMI); lateral condyle of femur (FLI); patellar groove of femur (FPI); medial plateaus of tibia covered by meniscus (TMI-M); medial plateaus of tibia uncovered by meniscus (TMI); lateral plateaus of tibia covered by meniscus (TLI-M); lateral plateaus of tibia uncovered by meniscus (TLI).

To obtain a better understanding of the mechanical nature of cartilage in different regions, great efforts have been given. Jurvelin et al. observed the distinct variations in elastic properties of canine femoral and tibial cartilage (FMI, FLI, FPI, TMI and TLI) and showed the stiffest cartilage is FPI and the softest one is TLI^2^. But in another paper, the authors reported that the softest cartilage is TMI^3^. Both Thambyah et al.^4^ and Setton et al.^5^ suggested the presence of meniscus results in a stiffer underneath cartilage in contrast to the absence of meniscus (TMI-M vs. TMI, TLI-M vs. TLI). Clearly, discrepancies exist, which may be ascribed to the differences in animal species, experimental methods and calculation methods^6,7^. As such, the region-dependent mechanical behavior of cartilage in knee joint has not be fully established so far.

Here, the mechanical properties of tibiofemoral and patellofemoral articular cartilages are discussed in terms of both region dependence (namely FMI, FLI, FPI, TMI-M, TMI, TLI-M, TLI) and strain rate dependence. In addition, based on the histological analyses, the relationship between the macro mechanical properties, biological structures and physiological function of different regions of cartilage have been discussed. Based on experimental data, the mechanical constitutive models of cartilage are also proposed, which could provide reference for numerical simulation to predict the cartilage damage and assessing cartilage replacement materials.

## Materials and methods

### Specimen preparation

**Figure 1:**
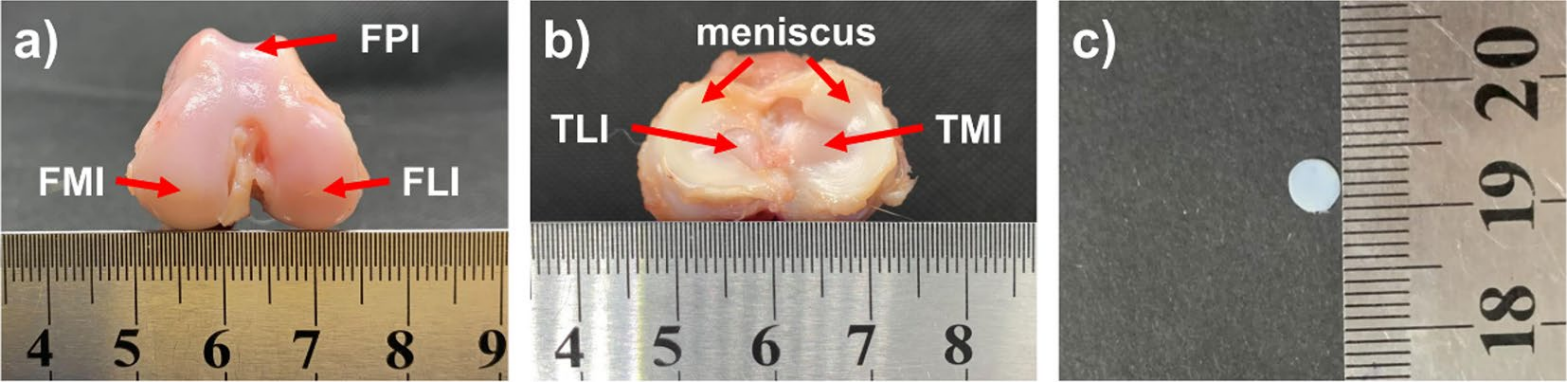
The cartilage samples for unconfined compression tests. The test regions in femur (**a**) and tibia (**b**). (**c**) The photo of cartilage disk samples.

Beagle dogs were used as experimental animals provided by Dalian Medical University (age: 3 years old, weight: 8–10 kg, raised in Experimental Animal Center of Dalian Medical University). After euthanasia, the hind limbs with all skin and connected tissue intact were packed in double plastic bags and frozen at − 20 °C. Prior to sample preparation, the intact joints were thawed at room temperature for 12 h^8,9^. After removing the surrounding muscle, the femur, tibia and patella were separated and immersed in physiological saline (0.9 wt.% sodium chloride aqueous solution) to prevent dehydration. The cylindrical cartilage disks with the diameter of 4 mm were cut with biopsy punch and scalpel from seven regions, i.e. FMI, FLI, FPI, TMI-M, TMI, TLI-M, TLI, as shown in Fig. 1a, b. Figure 1c gives the photo of one cartilage specimen. All the procedures were approved by the Bioethics Committee of Dalian University of Technology and performed in accordance with relevant guidelines and regulations. The animal study was carried out in compliance with the ARRIVE guidelines.

### Uniaxially unconfined compression test

Since the native cartilage is predominantly subjected to compression^10^, the uniaxially unconfined compression tests were performed on MTS Universal Testing Machine (MTS Criterion Model 43, MTS Systems Corp, USA). Total 12 knee joints from 12 dogs were used for the mechanical tests. The knee joints are randomly allocated to three groups for the strain rate of 3%/min, 30%/min, and 300%/min (n = 4 in each strain rate). From each joint, one sample was taken from each defined region without calcified cartilage and subchondral bone. The prepared cartilage samples were compressed to 0.6 strain along the axial direction (normal to the articular surface). The original thickness measurement and preloading were referred to the method of Williams et al.^11^. The thickness of cartilage was measured: FMI: 0.78 ± 0.13 mm, FLI: 0.47 ± 0.11 mm, FPI: 0.49 ± 0.14 mm, TMI-M: 0.68 ± 0.14 mm, TLI-M: 0.67 ± 0.18 mm, TMI: 1.10 ± 0.19 mm, TLI: 1.01 ± 0.20 mm (n = 12). The 0.2 N preload was applied to make the sample tightly contact with the upper and lower platens to approximate the plane compression. Prior to testing, the top and bottom platforms were lubricated with physiological saline to reduce friction between the contact surfaces. Only one test was executed on each sample.

### Histological analysis

The specimens from different regions were fixed in 10% buffered formalin overnight and then embedded in paraffin. The sections were cut into slices with the thickness of 5 µm using a microtome (RM2235, Leica, Germany) and deparaffinized for staining. Sections from each region were stained with Hematoxylin and Eosin (H&E), Safranin O-fast green, Toluidine blue and Sirius red respectively. The slices were observed under a microscope (IX83, Olympus, Japan) to investigate the differences in physiological structure and composition.

### Quantitative biochemical assays

Collagen and proteoglycans (PGs) are the major components of cartilage matrix. PGs are produced and modified by glycosaminoglycan (GAG) chains in the secretory pathway of animal cells^12^, thus the content of GAG was determined to reflect the content of PGs. GAG contents were determined using a dimethylmethylene blue (DMMB) assay. The cartilage tissue isolated from different regions were digested with papain solution (0.25 mg/mL in 0.2 mol/L sodium bicarbonate, 0.01 mol/L EDTA, and 5 mmol/L L-cysteine at 60 °C overnight, Solarbio, China). The digest solution was used with Blyscan GAG Assay (Biocolor, UK), and the absorbance was read at 656 nm. Quantification of collagen was based on the hydroxyproline content of the samples^13^. Cartilage samples were hydrolyzed by 6 mol/L HCl at 100 °C for 5 h. The hydroxyproline content was determined by the hydroxyproline assay kit (Nanjing Jiancheng Bioengineering Institute, China), and the absorbance was recorded at 550 nm. These biochemical quantification tests were repeated three times.

### Constitutive model fitting

Assuming articular cartilage as an isotropic incompressible material, three widely used isotropic hyperelastic constitutive models were tested to characterize cartilage behavior, i.e. the Fung, Gent and Ogden model^14^. According to the study about similar biological tissue^15,16^, the engineering stress among the compression direction $\sigma_{1}$ can be calculated using the following equations.

1. In Fung model^17^
  1 $$\sigma_{1} = \mu_{0} e^{{b\left( {\lambda_{1}^{2} + 2\lambda_{1}^{ - 1} - 3} \right)}} \left( {\lambda_{1} - \lambda_{1}^{ - 2} } \right)$$
2. In Gent model^18^
  2 $$\sigma_{1} = \frac{{\mu_{0} J_{m} \left( {\lambda_{1} - \lambda_{1}^{ - 2} } \right)}}{{J_{m} - \lambda_{1}^{2} - 2\lambda_{1}^{ - 1} + 3}}$$
3. In Ogden model^19^
  3 $$\sigma_{1} = \frac{{2\mu_{0} }}{\alpha }\left[ {\lambda_{1}^{\alpha - 1} - \lambda_{1}^{{ - \left( {\frac{\alpha }{2} + 1} \right)}} } \right]$$
  where the stretch ratio λ is calculated from the engineering strain ε using equation: λ = 1 + ε. In these equations, $\mu_{0}$ and *b*, $J_{m}$, $\alpha$ are constant coefficients obtained in experimental curve fitting, $\mu_{0}$ is the initial shear modulus and *b*, $J_{m}$, $\alpha$ are the stiffening parameters. The hyperelastic constitutive fitting of Fung, Gent and Ogden models was performed using MATLAB R2014a for the mean stress–strain curves at strain rate 3%/min, 30%/min and 300%/min.

The accuracy of curve fitting was evaluated using nMSE (normalized mean square error), which represented the overall deviations between the experimental data and fitted data. The lower nMSE value signifies the better fitting of model. nMSE was determined as the equation:

4 $${\text{nMSE}} = \frac{{\sum {\left\| {\hat{y} - y} \right\|^{2} } }}{{\sum {\left\| y \right\|^{2} } }}$$

where $\hat{y}$ is the stress of fitted curves, and ${\text{y}}$ is the stress of experimental data.

### Statistical analysis

All the experimental data were expressed as mean ± SD. The statistical analysis was used one-way ANOVA analysis in SPSS (SPSS software 20.0) to determine the significant difference between groups.

## Results

### Uniaxially unconfined compression

#### Effect of strain rate on compressive properties

**Figure 2:**
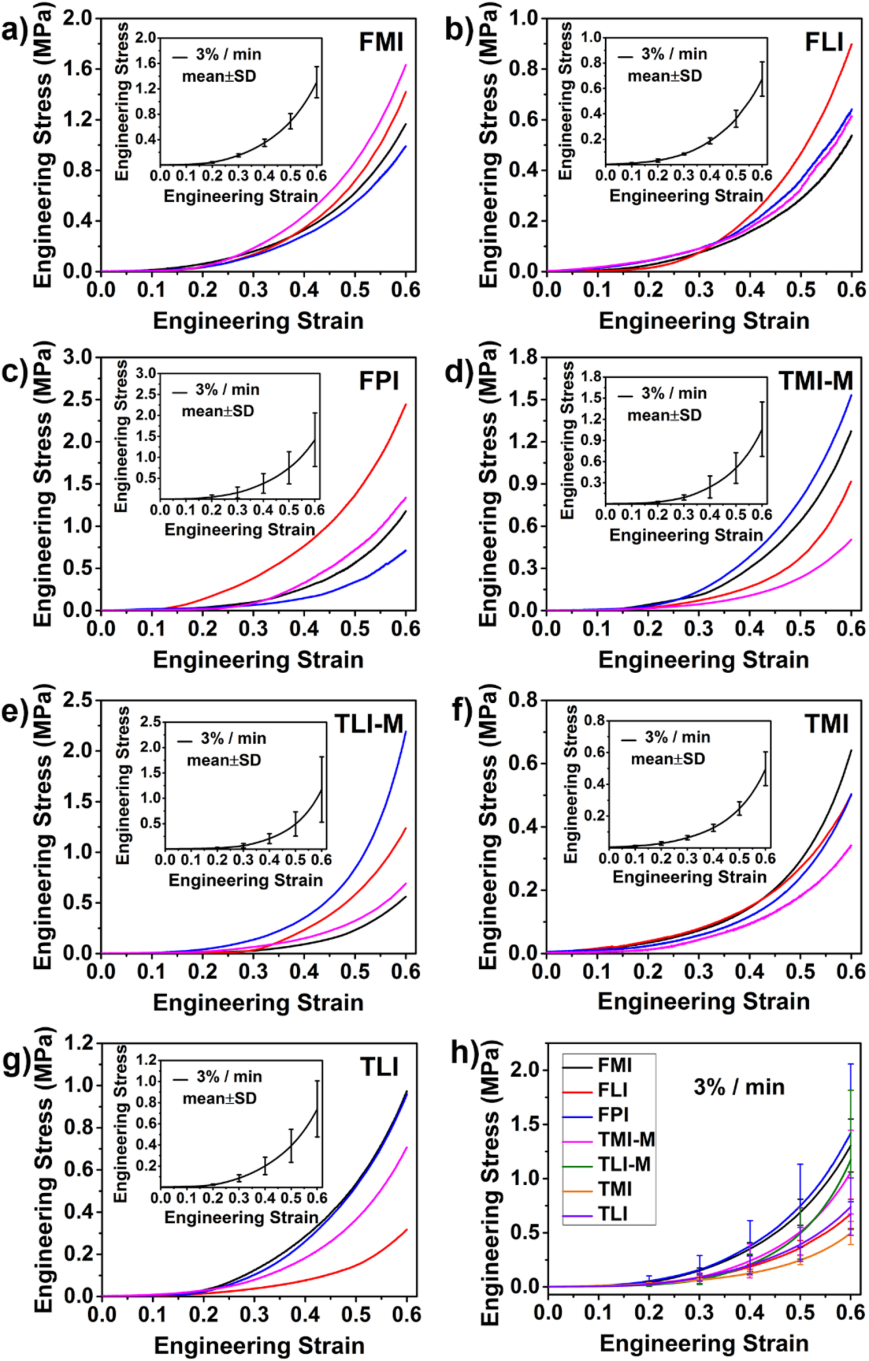
(**a**–**g**) Stress–strain curves of cartilage in predetermined regions at strain rate 3%/min. Inset: mean of four repetitions with error bar (± SD). (**h**) The comparison of mean stress–strain curves for seven regions at strain rate 3%/min.

**Figure 3:**
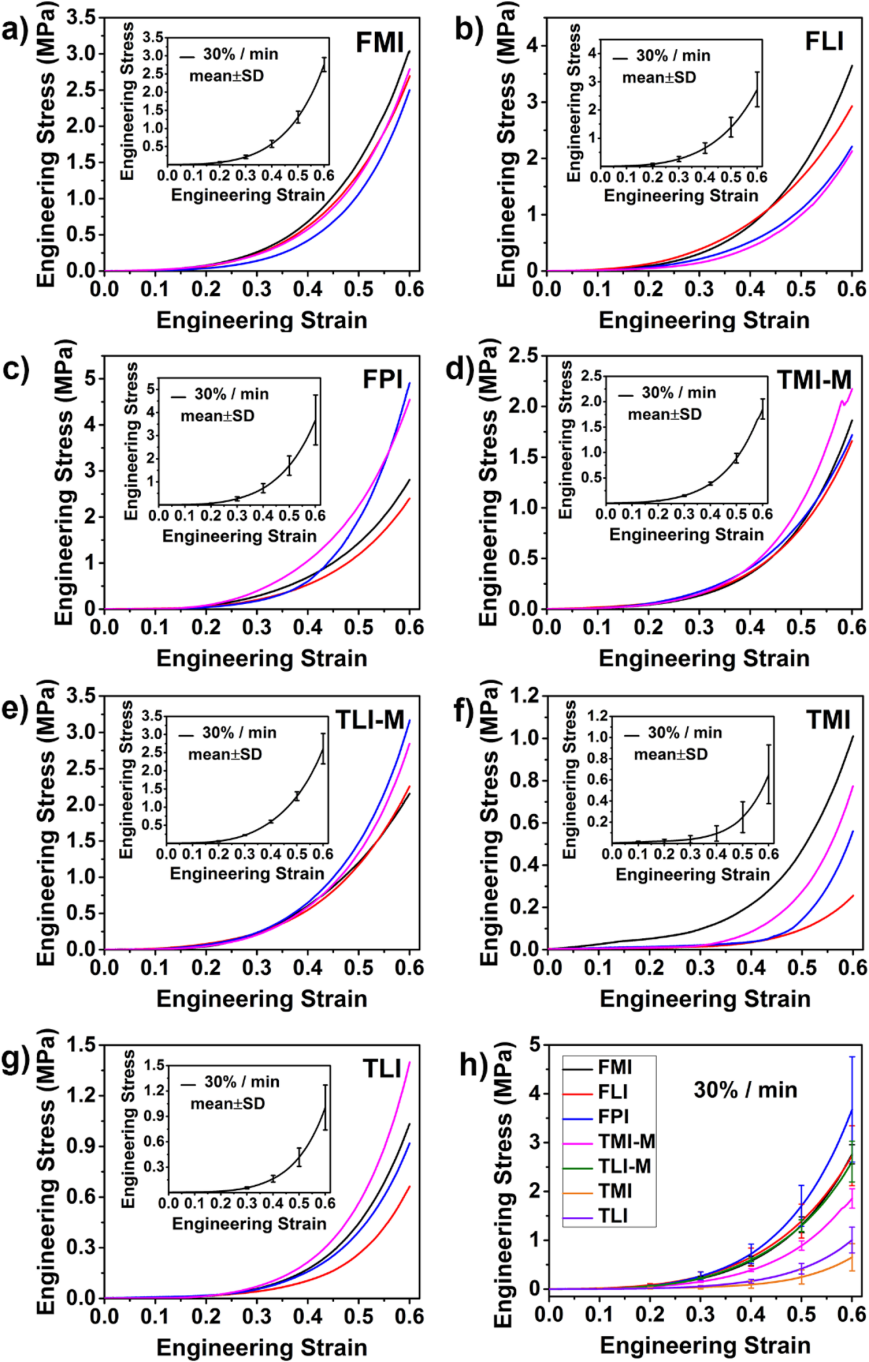
(**a**–**g**) Stress–strain curves of cartilage in predetermined regions at strain rate 30%/min. Inset: mean of four repetitions with error bar (± SD). (**h**) The comparison of mean stress–strain curves for seven regions at strain rate 30%/min.

**Figure 4:**
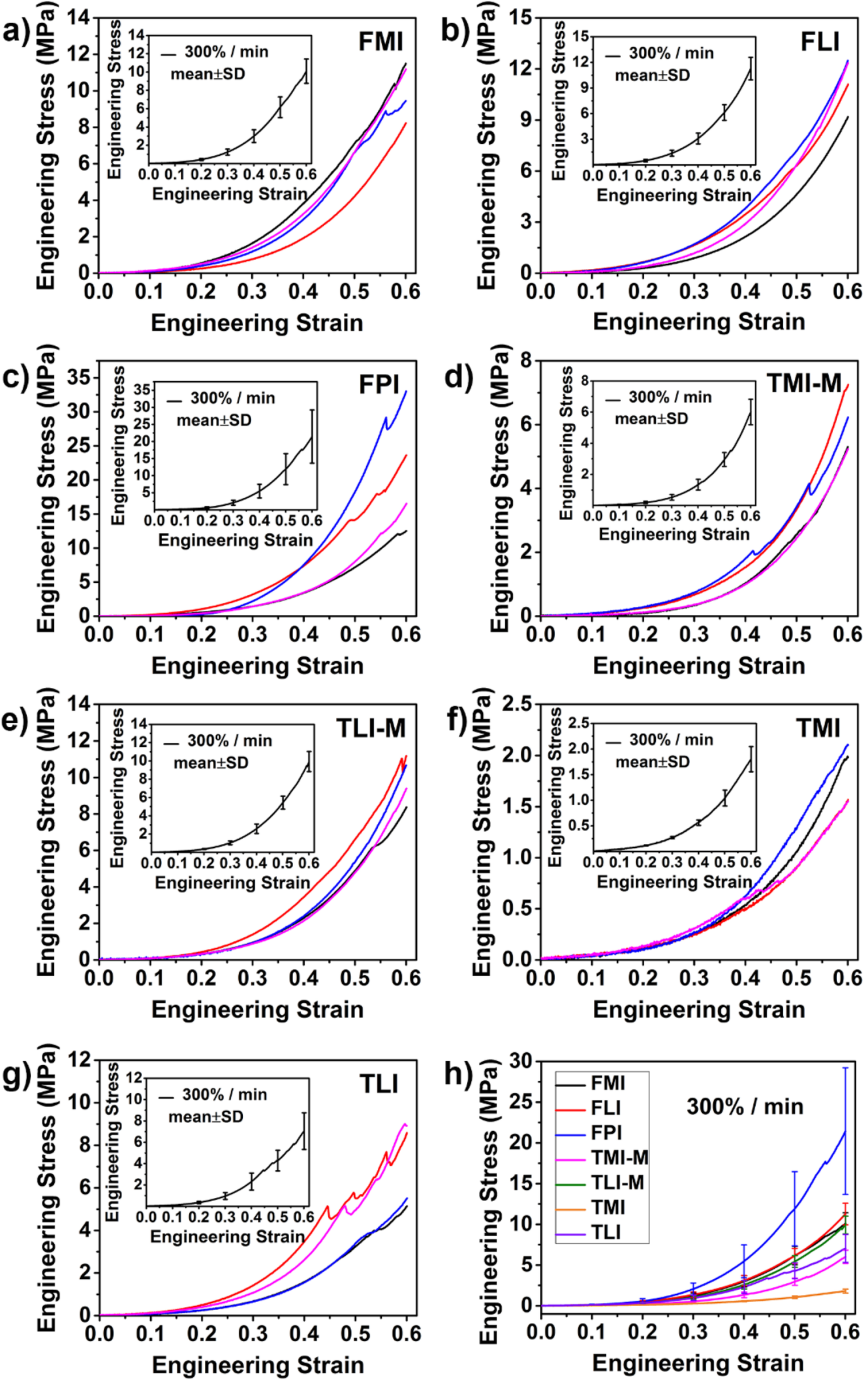
(**a**–**g**) Stress–strain curves of cartilage in predetermined regions at strain rate 300%/min. Inset: mean of four repetitions with error bar (± SD). (**h**) The comparison of mean stress–strain curves for seven regions at strain rate 300%/min.

The engineering stress–strain curves of articular cartilage in compression at strain rate of 3%, 30% and 300%/min are shown in Figs. 2, 3, 4 respectively, and the corresponding insets give the mean of four repetitions with error bars (mean ± SD). As shown in Figs. 2, 3, 4, these curves of cartilage in all regions show typical concave nonlinear characteristics, indicating the viscoelastic property of cartilage. At small strain, the compressive stress increases gently, but with increasing strain, the level of increase in stress gradually accelerates. The compressive moduli of cartilage at different regions at strain rate of 3%, 30% and 300%/min are presented in Table 1. The compressive modulus at each strain is defined here as the tangent of the stress–strain curves. In the initial strain range (0–0.2 strain), the compressive modulus is small and the increase amplitude is also slight. As the test continues, the compressive modulus substantially increases.

**Table 1:** Compressive modulus (MPa, mean ± SD) at strain rate 3%/min, 30%/min and 300%/min.

Strain rate	Strain	Femur			Tibia			
		FMI	FLI	FPI	TMI-M	TLI-M	TMI	TLI
3%/min	0.1	0.20 ± 0.07	0.14 ± 0.08	0.34 ± 0.26	0.12 ± 0.03	0.17 ± 0.13	0.14 ± 0.06	0.10 ± 0.03
	0.2	0.71 ± 0.13	0.35 ± 0.01	0.73 ± 0.69	0.43 ± 0.16	0.33 ± 0.26	0.26 ± 0.05	0.40 ± 0.16
	0.3	1.40 ± 0.26	0.71 ± 0.14	1.48 ± 0.93	0.93 ± 0.48	0.74 ± 0.32	0.43 ± 0.05	0.81 ± 0.36
	0.4	2.57 ± 0.50	1.34 ± 0.34	2.79 ± 1.28	1.95 ± 0.90	1.91 ± 0.88	0.89 ± 0.12	1.48 ± 0.62
	0.5*	4.53 ± 0.90	2.35 ± 0.56	4.98 ± 1.90	3.83 ± 1.36	4.43 ± 2.37	1.72 ± 0.38	2.56 ± 0.91
	0.6	7.59 ± 1.55	3.84 ± 0.81	8.35 ± 3.13	6.91 ± 1.97	8.90 ± 5.44	3.26 ± 0.91	4.20 ± 1.19
30%/min	0.1**	0.31 ± 0.07	0.36 ± 0.13	0.32 ± 0.06	0.24 ± 0.03	0.22 ± 0.12	0.10 ± 0.09	0.10 ± 0.02
	0.2**	0.94 ± 0.18	1.14 ± 0.40	1.18 ± 0.50	0.62 ± 0.08	1.30 ± 0.48	0.13 ± 0.11	0.24 ± 0.06
	0.3***	2.24 ± 0.44	2.60 ± 0.78	2.86 ± 0.86	1.48 ± 0.15	2.44 ± 0.16	0.33 ± 0.25	0.58 ± 0.16
	0.4***	5.08 ± 0.62	5.33 ± 1.34	6.65 ± 1.66	3.43 ± 0.45	4.77 ± 0.64	0.87 ± 0.56	1.62 ± 0.45
	0.5***	10.31 ± 0.62	9.92 ± 2.25	13.85 ± 4.52	7.04 ± 1.08	9.40 ± 1.61	2.51 ± 1.05	3.86 ± 1.02
	0.6**	18.81 ± 1.06	16.97 ± 3.72	25.75 ± 10.21	12.92 ± 2.15	16.72 ± 4.45	5.88 ± 2.13	7.84 ± 2.06
300%/min	0.1	1.28 ± 0.61	2.11 ± 0.65	2.46 ± 0.68	0.81 ± 0.56	1.24 ± 0.17	0.48 ± 0.14	0.95 ± 0.32
	0.2**	4.61 ± 1.79	5.76 ± 1.57	8.23 ± 3.89	1.96 ± 1.18	4.12 ± 1.16	1.04 ± 0.25	3.97 ± 1.97
	0.3***	12.79 ± 3.11	12.24 ± 2.59	19.85 ± 8.42	4.62 ± 1.55	10.57 ± 2.26	2.12 ± 0.33	9.79 ± 3.19
	0.4***	23.82 ± 4.33	22.89 ± 3.42	37.75 ± 11.85	10.31 ± 2.16	20.95 ± 2.74	3.79 ± 0.86	16.79 ± 4.02
	0.5***	35.68 ± 3.51	39.07 ± 4.52	62.35 ± 13.04	20.55 ± 4.50	35.61 ± 3.85	6.16 ± 1.21	23.37 ± 5.15
	0.6***	51.47 ± 2.60	62.13 ± 7.85	94.05 ± 17.46	36.87 ± 10.35	54.93 ± 10.05	9.33 ± 1.79	27.90 ± 8.01

Significant different between different cartilage regions: *p < 0.05, **p < 0.01, ***p < 0.001.

Figures 2, 3 and 4 also shows the stress–strain curves under different loading rates exhibit the strain rate dependent behavior. Upon the same loads, the deformation of cartilage decreases with the increase of strain rate. Taking FMI as an example, when loaded to 1 MPa, the strain reaches 0.56 at 3%/min strain rate and 0.47 at 30%/min strain rate, while the strain only reaches 0.28 at the strain rate of 300%/min. This trend is true for all the regions of cartilage, the compressive modulus increases with strain rate, presenting a "stiffening" appearance.

#### Effect of cartilage regions on compressive properties

As shown in Figs. 2, 3, 4 and Table 1, the regional variation of mechanical properties is apparent. Firstly, the differences of compressive properties can be observed in the femoral and tibial cartilage. There is a relatively higher deformation in the tibial cartilage than the femoral cartilage at the same loading. Moreover, the same trend is observed in the compressive moduli shown in Table 1. In general, the femoral cartilage has a higher stiffness than the tibial cartilage. For the femoral cartilage, there is no considerable difference in the compressive properties between the medial and lateral femoral condyles (FMI and FLI). Yet, the stiffest cartilage is found in patellar groove of the femur (FPI).

For tibial cartilage, it is essential to consider the effect of meniscus^20^. Hence, the tibial cartilage is divided into four regions depending on the absence/presence of meniscus. As seen in Table 1, the compressive modulus at 0.1 strain is 0.81 ± 0.56 MPa for TMI-M while 0.48 ± 0.14 MPa for TMI when strain rate is 300%/min, suggesting the cartilages of tibial plateau covered by the meniscus (TMI-M and TLI-M) are stiffer compared with that not covered by the meniscus (TMI and TLI).

### Biochemical analysis

**Figure 5:**
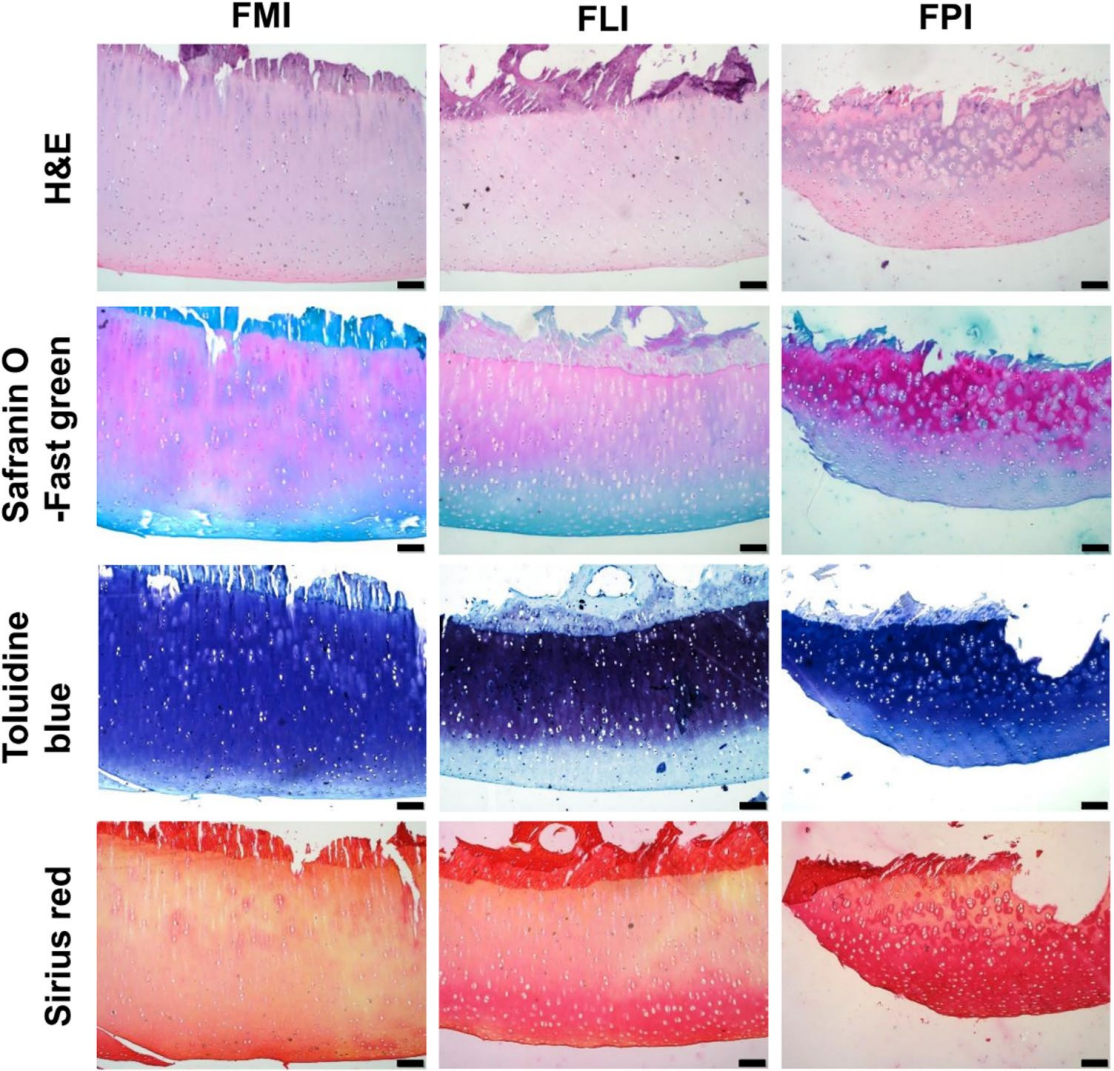
Histological analysis of specific regions in femoral cartilage using H&E, Safranin O-fast green, Toluidine blue and Sirius red staining. Scale bar: 100 μm.

**Figure 6:**
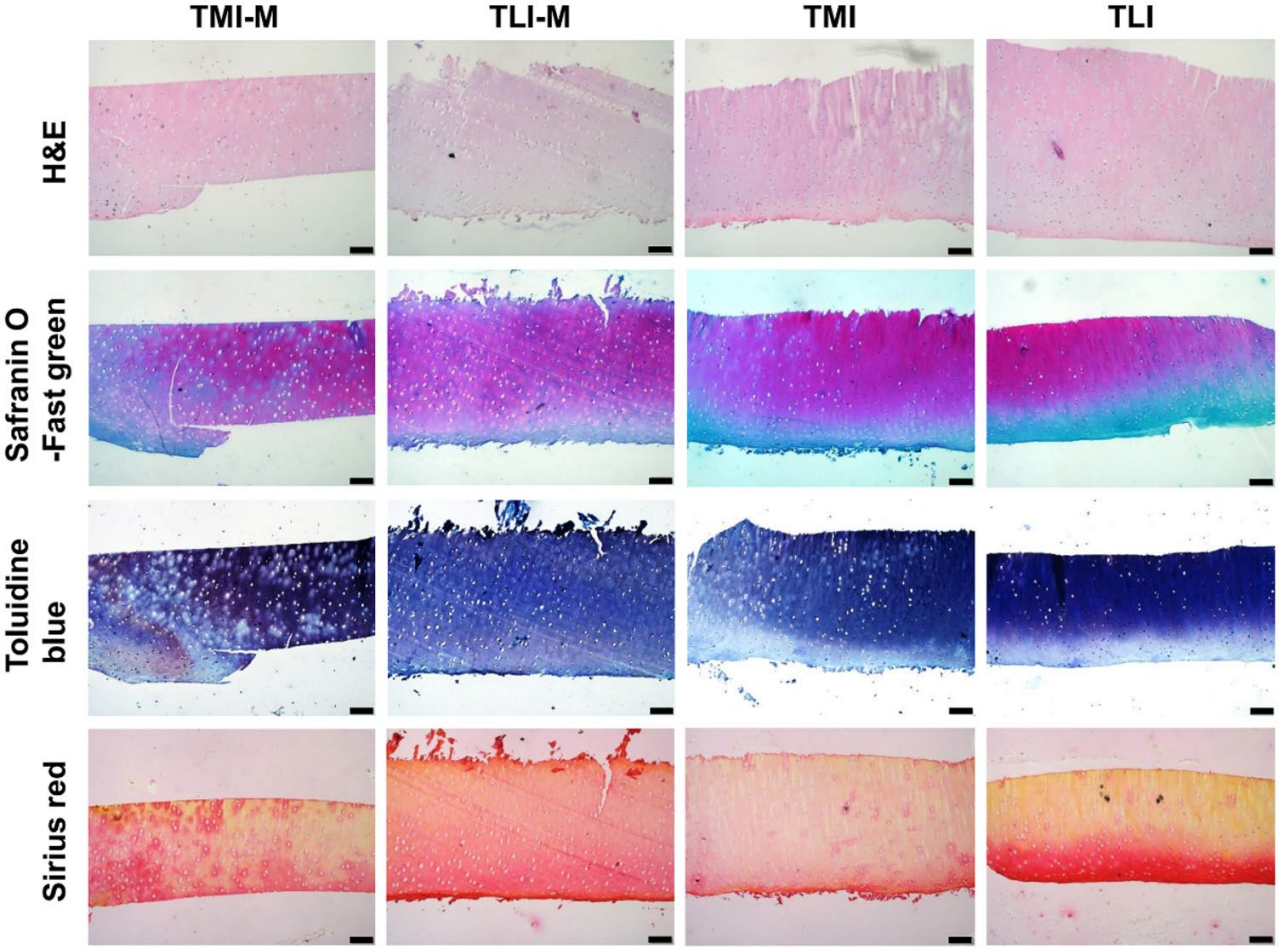
Histological analysis of specific regions in tibial cartilage using H&E, Safranin O-fast green, Toluidine blue and Sirius red staining. Scale bar: 100 μm.

The histological staining is conducted to characterize the structures and compositions, and the results are given in Figs. 5 and 6. H&E staining indicates, the surface of cartilage is smooth and has the hierarchical structure. Histological sections stained with Safranin O-fast green could dye PGs red and the chondrocyte nuclei green. The rounded chondrocytes can be observed and the four-layer structure is more distinct, which is in agreement with H&E staining.

Toluidine blue staining is used to detect the distribution and content of PGs in cartilage matrix showing as blue-purple. It can be observed that the superficial layer is slightly stained, and the middle and deep layers are deeper staining, demonstrating the hierarchical structure of cartilage. This phenomenon is visible in all the seven cartilage regions. Although previous studies have shown that the content of PGs in tibial cartilage is higher than in femoral cartilage^21^, uneven staining and local loss of staining are observed in Fig. 6, indicating the higher content but inhomogeneous distribution of PGs in tibial cartilage.

The collagen is examined by Sirius red staining, in which collagen fibers are stained red and the nuclei of chondrocytes are yellow. The collagen content varies with cartilage depth, being highest in the surface zone and decreasing in the middle and deep zones, which is contrary to variation of PGs content. All regions of articular cartilage are positive for Sirius red staining. Among all regions, patellar groove of femur and femoral condyles which are subjected to high shear stress in physiological activities, are deeper stained and show the higher collagen content than the tibial plateaus preferentially subjected to compressive load^22^. Furthermore, large area is negative stained in the middle and deep layers of TMI and TLI, implying the lower collagen content.

**Figure 7:**
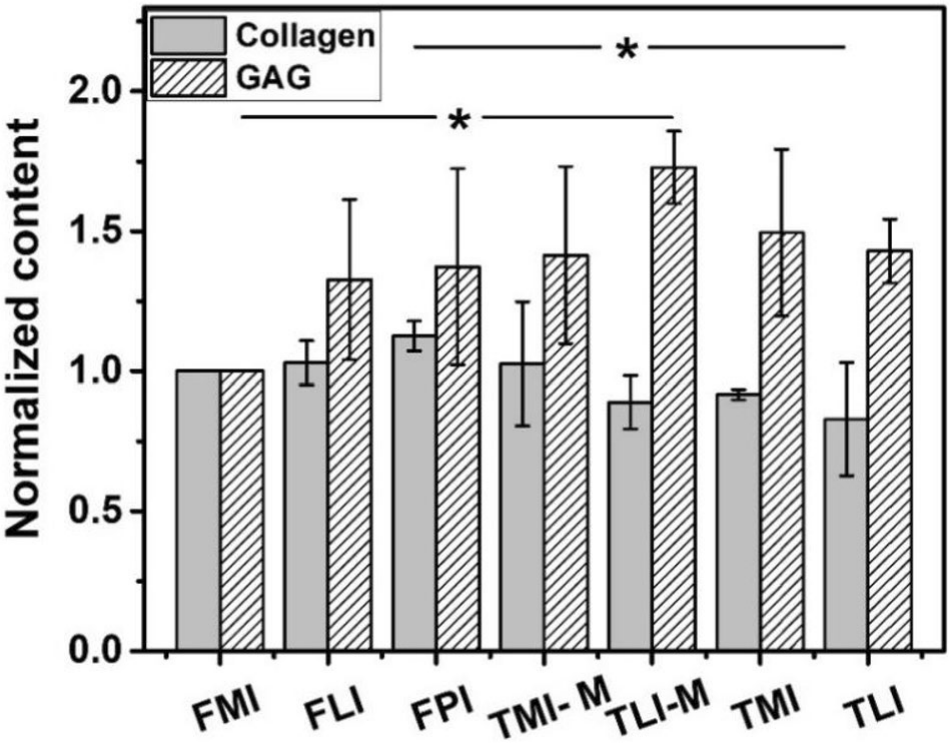
The normalized content of cartilage components in predefined regions (n = 3, *p* = 0.032 for collagen, *p* = 0.010 for GAG).

To further investigate the biochemical components of cartilage in different regions, the GAG and hydroxyproline contents in samples are examined to reflect the contents of PGs and collagen, respectively. The normalized results (taking FMI as the standard) are shown in Fig. 7, and the original data are presented in Table S1 (see Supplementary Materials). Figure 7 indicates that the content of PGs in tibial cartilage is higher than in femoral cartilage. For collagen, it can be seen that in the seven regions investigated, the cartilage on the FPI shows the highest collagen content, and TLI shows the lowest collagen content, which agrees well with the histological analyses.

### Constitutive model fitting

The hyperelastic constitutive fittings of Fung, Gent and Ogden models are performed for the mean stress–strain curves at strain rate 3%/min, 30%/min and 300%/min. The fitting results are shown in Fig. S1–3 (see Supplementary Materials). At different strain rates, the fitting curves of the three models at different regions of articular cartilage are well fitted with the experimental data. The material parameters and nMSE values of the three models are listed in Table 2. It is clear that for three models, the overall deviations between fitted and tested data are all lower than 0.03, indicating the good fitting. It can be seen from these fitting parameters that the initial shear modulus $\mu_{0}$ of three models increases monotonically with the strain rate increasing. Furthermore, the stiffening parameters *b* in Fung model and $\alpha$ in Ogden model also show a monotonically increasing trend with the strain rate, while the stiffening parameter $J_{m}$ in Gent model decreases first and then increases.

**Table 2:** Material parameters for cartilage obtained by fitting the models to experimental data.

	Strain rate (min^−1^)	Fung			Gent			Odgen		
		$\mu_{0}$	*b*	nMSE	$\mu_{0}$	$J_{m}$	nMSE	$\mu_{0}$(e^−2^)	$\alpha$	nMSE
FMI	3%	0.13	2.62	0.004	0.24	1.01	0.020	3.82	12.56	0.005
	30%	0.25	2.69	0.007	0.43	0.97	0.025	6.66	12.91	0.000
	300%	1.01	2.87	0.010	2.17	1.05	0.026	25.01	13.24	0.016
FLI	3%	0.08	2.52	0.003	0.13	1.02	0.017	3.61	10.84	0.000
	30%	0.27	2.65	0.005	0.47	0.99	0.023	9.66	11.94	0.001
	300%	1.10	2.77	0.005	2.19	1.02	0.019	26.95	13.17	0.010
FPI	3%	0.15	2.64	0.004	0.26	1.01	0.019	6.66	11.21	0.001
	30%	0.30	2.75	0.006	0.55	0.96	0.030	7.71	13.26	0.000
	300%	1.88	2.91	0.009	4.05	1.01	0.031	37.46	14.07	0.013
TMI-M	3%	0.08	3.03	0.003	0.17	0.98	0.022	3.14	12.36	0.000
	30%	0.13	3.11	0.004	0.30	0.98	0.025	4.60	12.87	0.000
	300%	0.41	3.14	0.003	0.97	0.98	0.021	12.81	13.29	0.002
TLI-M	3%	0.08	3.07	0.006	0.16	0.94	0.026	3.53	12.07	0.007
	30%	0.17	3.15	0.005	0.41	0.99	0.026	7.32	12.35	0.001
	300%	0.70	3.20	0.011	1.87	1.01	0.024	25.94	12.91	0.006
TMI	3%	0.05	2.60	0.001	0.09	1.01	0.010	2.19	11.54	0.009
	30%	0.05	2.72	0.020	0.08	0.94	0.022	2.27	11.81	0.029
	300%	0.18	2.81	0.009	0.40	1.06	0.014	6.99	11.97	0.009
TLI	3%	0.08	2.65	0.004	0.14	1.02	0.020	3.62	11.08	0.001
	30%	0.09	2.66	0.018	0.13	0.94	0.027	3.91	11.29	0.018
	300%	0.75	2.71	0.015	1.63	1.08	0.025	31.08	11.64	0.012

*The unit of $\mu_{0}$ is MPa.

## Discussion

Cartilage is a biphasic material composed of a mass of interstitial fluid and the solid matrix, it usually exhibits a typical viscoelastic behavior when subjected to mechanical loading. The quasi-static unconfined compression tests were conducted and discussed in this study. With the initial compression loading, the interstitial fluid in cartilage matrix flows out relatively quickly, and the compressed cartilages are prone to deform, showing the gentle increasing of compressive stress and modulus. With the increasing loading, the resistance of fluid flow increases. The fluid pressure in the matrix maintained by the PGs and collagen network increases and enhancing the ability to bear external loading, so the cartilage presents a stiffer characteristic^23,24^.

The strain rate dependence is also ascribed to the biphasic structure of cartilage. As mentioned above, the deformation of cartilage is mainly due to the outflow of interstitial fluid. At high strain rates, the fluid has little time to dissipate and the resistance of fluid flow increases, generating a higher hydrostatic pressure to resist compression loads^21,25^. At this point, cartilage tends to show stronger resistance to pressure.

As stated, the mechanical properties of the cartilage in tibiofemoral joint and patellofemoral joint show distinct regional variation, which may be relevant to the physiology locomotion of knee joint and the load transmitting among joint surfaces. The stiffest cartilage is found in FPI, where is regarded as the frequent contacting area in knee joint. Under physiological loading, contact stresses for the femoral groove are larger than that for the femoral condyle^4^. It can be understood that the higher compressive stiffness for FPI could reflect the physiological requirement. For tibial cartilage, compression test results indicate that TMI-M and TLI-M are stiffer than TMI and TLI. This can be explained by the difference in the physiological loading environment. The tibial cartilage not covered by meniscus directly bears loads, and the soft but thick cartilage (TMI and TLI) provides a large contact area during loading, improving the shock absorption ability and protecting cartilage and bone from excessive loads. For the tibial cartilage covered by meniscus, the loads transmit through meniscus to the cartilage and the meniscus can dissipate stress and absorb shock, resulting in the stiff but thin cartilage (TMI-M and TLI-M).

The properties of biological tissue appear to be conditioned to the functional requirement in physiological activities, and its composition and structure determine the macroscopic mechanical properties. Through histological staining and quantitative biochemical assay, the cartilage structure, composition content and distribution have been examined. The biological differences in different cartilage regions and the relationship with mechanical performances will be discussed.

When articular cartilage is compressed, collagen-proteoglycans matrix and interstitial fluid interact in a unique manner to resist stress^26^. Collagen network provides superior tensile property and restricts the expansion of PGs to control the instant shape change. In addition, PGs carry the negative charges, and due to the charge repulsion effect, the osmotic expansion pressure is formed. When the cartilage is stressed, the flow of interstitial fluid causes the increase of the osmotic expansion pressure to against external stress^21,27^. As discussed above, the tibia cartilage is rich in PGs but relatively poor in collagen network. In TMI, the separation of PGs is observed, and the collagen content is low and the fibers are apart from the PGs. It is difficult for the collagen network to provide resistance against the deformation of cartilage matrix while loaded and maintain a stable osmotic pressure to resist the applied stress, which makes TMI the softest region in the cartilage of whole joint. Likewise, the lateral condyle of tibial cartilage has the similar properties. On the contrary, FPI has a high concentration of collagen, the fiber bundles are closely arranged, and no single fiber can be observed. Meanwhile, toluidine blue staining shows that the PGs are evenly distributed. Based on this composition and structure analyses, it is reasonable to postulate that the tightly aggregated collagen networks restrict the deformation of the PGs, while the entrapped PGs provide resistance against the movement of the collagen network by intermolecular frictional and steric exclusion effects. This mechanism contributes to the liquid pressurization in cartilage matrix, which is the main cause of the high compression stiffness in FPI.

The Fung, Gent and Ogden models are generally accepted for describing the mechanical behavior of articular cartilage^28,29^, thus these models are used to fit our curves with the attempt to give the constitutive model of articular cartilage at quasi-static compression. As the fitting results shown in Table 2, the nonlinear behavior was modeled well by Fung, Gent and Ogden hyperelastic models. Considering the stiffening effect of the viscoelastic biological tissues incurred by strain rate, the stiffening parameters should show a monotonically increase with strain rates^15,30^. Therefore, the Fung and Ogden models seem more qualified to represent the mechanical behavior of cartilage during compression. The nonlinear, region dependent, and strain rate dependent mechanical behavior can all be modeled well by Fung and Ogden hyperelastic models, which could provide reference for numerical simulation to predict the cartilage damage and assessing cartilage replacement materials.

There are some limitations in this study. (1) Due to the curved morphology of cartilage, though the preload is applied, it is hard to obtain the completely flat samples, which may result in the experimental errors. (2) The tangent modulus can reflect the elastic properties of cartilage, but it cannot represent the overall mechanical performance due to the viscoelastic property of cartilage. (3) Compared with hyperelastic model, the biphasic models may reflect the mechanical characteristics of cartilage thoroughly^31–33^. Thus, the relaxation or creep experiments need to be performed to determine the relative viscoelastic parameters and propose the biphasic model.

## Conclusion

In this study, the mechanical and biological properties of tibiofemoral and patellofemoral articular cartilages are investigated using unconfined compression test and histological sections. (1) The stress–strain behavior of cartilage in unconfined compression shows a typical non-linear trend, and the mechanical property depends on the compression loading rate. The compressive modulus increases with the increase of strain and also strain rate. (2) The cartilage exhibits region dependence. The femoral cartilage is stiffer than the tibial cartilage, and the cartilage in femoral groove (FPI) is stiffest in the knee joint. This may be ascribed to the liquid pressurization in cartilage matrix triggered by the tightly aggregated collagen networks and the entrapped PGs resulting the higher stiffness. The cartilages of tibial plateau covered by the meniscus (TMI-M and TLI-M) are stiffer compared with that not covered by the meniscus (TMI and TLI). The reason might be the lowest content of collagen and the separation of PGs in TMI and TLI hardly providing resistance against the deformation of cartilage and maintaining a stable osmotic pressure to resist loads. (3) The Fung’s and Ogden’s models could represent the stiffening effect incurred by the increase of strain rate during compression.

## Supplementary Information

Supplementary Information 1.

Supplementary Information 2.

## References

[1] Rai V, Dilisio MF, Dietz NE, Agrawal DK (2017). Recent strategies in cartilage repair: a systemic review of the scaffold development and tissue engineering. J. Biomed. Mater. Res. A.

[2] Jurvelin JS, Kiviranta I, Arokoski J, Tammi M, Helminen HJ (1987). Indentation study of the biomechanical properties of articular cartilage in the canine knee. Eng. Med..

[3] Jurvelin JS, Arokoski JPA, Hunziker EB, Helminen HJ (2000). Topographical variation of the elastic properties of articular cartilage in the canine knee. J. Biomech..

[4] Thambyah A, Nather A, Goh J (2006). Mechanical properties of articular cartilage covered by the meniscus. Osteoarthr Cartil.

[5] Setton LA, Mow VC, Müller FJ, Pita JC, Howell DS (1994). Mechanical properties of canine articular cartilage are significantly altered following transection of the anterior cruciate ligament. J. Orthop. Res..

[6] Athanasiou KA, Rosenwasser MP, Buckwalter JA, Malinin TI, Mow VC (2010). Interspecies comparisons of in situ intrinsic mechanical properties of distal femoral cartilage. J. Orthop. Res..

[7] Korhonen RK, Laasanen MS, Tyrs J, Rieppo J, Jurvelin JS (2002). Comparison of the equilibrium response of articular cartilage in unconfined compression, confined compression and indentation. J. Biomech..

[8] Schinagl RM, Gurskis D, Chen AC, Sah RL (1997). Depth-dependent confined compression modulus of full-thickness bovine articular cartilage. J. Orthop. Res..

[9] LeRoux MA (2000). Simultaneous changes in the mechanical properties, quantitative collagen organization, and proteoglycan concentration of articular cartilage following canine meniscectomy. J. Orthop. Res..

[10] Chan DD (2016). In vivo articular cartilage deformation: noninvasive quantification of intratissue strain during joint contact in the human knee. Sci. Rep..

[11] Williams LN, Elder SH, Bouvard JL, Horstemeyer MF (2008). The anisotropic compressive mechanical properties of the rabbit patellar tendon. Biorheology.

[12] Prydz K (2015). Determinants of glycosaminoglycan (GAG) structure. Biomolecules.

[13] Reddy GK, Enwemeka CS (1996). A simplified method for the analysis of hydroxyproline in biological tissues. Clin. Biochem..

[14] Wex C, Arndt S, Stoll A, Bruns C, Yuliya K (2015). Isotropic incompressible hyperelastic models for modelling the mechanical behaviour of biological tissues: a review. Biomed. Eng. Biomed. Tech..

[15] Zhang W (2016). Mechanical response of brain stem in compression and the differential scanning calorimetry and FTIR analyses. J. Appl. Mech. Trans. ASME.

[16] Li Y, Zhang W, Lu YC, Wu CW (2020). Hyper-viscoelastic mechanical behavior of cranial pia mater in tension. Clin. Biomech.

[17] Fung YC (1967). Elasticity of soft tissues in simple elongation. Am. J. Physiol..

[18] Gent AN (1996). A new constitutive relation for rubber. Rubber Chem. Technol..

[19] Ogden RW (1972). Large deformation isotropic elasticity - on the correlation of theory and experiment for incompressible rubberlike solids. Proc. R. Soc. A Math. Phys. Eng. Sci..

[20] Danso EK (2017). Structure-function relationships of human meniscus. J. Mech. Behav. Biomed. Mater..

[21] Jurvelin J, Säämänen AM, Arokoski J, Helminen HJ, Tammi M (1988). Biomechanical properties of the canine knee articular cartilage as related to matrix proteoglycans and collagen. Eng. Med..

[22] Arokoski JPA, Jurvelin JS, Väätäinen U, Helminen HJ (2000). Normal and pathological adaptations of articular cartilage to joint loading. Scand. J. Med. Sci. Sports Rev. Article.

[23] Li LP, Herzog W (2004). Strain-rate dependence of cartilage stiffness in unconfined compression: the role of fibril reinforcement versus tissue volume change in fluid pressurization. J. Biomech..

[24] Li LP, Buschmann MD, Shirazi-Adl A (2003). Strain-rate dependent stiffness of articular cartilage in unconfined compression. J. Biomech. Eng..

[25] Burgin LV, Edelsten L, Aspden RM (2014). The mechanical and material properties of elderly human articular cartilage subject to impact and slow loading. Med. Eng. Phys..

[26] Julkunen P (2009). Biomechanical, biochemical and structural correlations in immature and mature rabbit articular cartilage. Osteoarthr. Cartil..

[27] Shirazi R, Shirazi-Adl A, Hurtig M (2008). Role of cartilage collagen fibrils networks in knee joint biomechanics under compression. J. Biomech..

[28] Lin DC (2009). Spherical indentation of soft matter beyond the Hertzian regime: numerical and experimental validation of hyperelastic models. Biomech. Model. Mech..

[29] Khajehsaeid H, Abdollahpour Z (2020). Progressive deformation-induced degradation of knee articular cartilage and osteoarthritis. J. Biomech..

[30] Rashid B, Destrade M, Gilchrist MD (2012). Mechanical characterization of brain tissue in compression at dynamic strain rates. J. Mech. Behav. Biomed. Mater..

[31] Mow VC (1980). Biphasic creep and stress relaxation of articular cartilage in compression: theory and experiments. J. Biomech. Eng..

[32] Huang CY, Mow VC, Ateshian GA (2001). The role of flow-independent viscoelasticity in the biphasic tensile and compressive responses of articular cartilage. J. Biomech. Eng..

[33] Wilson W (2005). A fibril-reinforced poroviscoelastic swelling model for articular cartilage. J. Biomech..

