# Supplementary material for: The mechanical properties of tibiofemoral and patellofemoral articular cartilage in compression depend on anatomical regions
Source: Sci Rep. 2021 Mar 17;11:6128. doi: 10.1038/s41598-021-85716-2 (PMC7969630; doi:10.1038/s41598-021-85716-2)
Supplement: Supplementary file 2 — Supplementary Information 2. [file 41598_2021_85716_MOESM2_ESM.docx]

**Supplementary Materials**

The mechanical properties of tibiofemoral and patellofemoral articular cartilage in compression depend on anatomical regions

Heng Li*^a^*, Jinming Li*^a^*, Shengbo Yu*^b^*, Chengwei Wu*^a^*, Wei Zhang*^a,^*^^[[1]](#footnote-1)^*^

*^a^* State Key Laboratory of Structure Analysis for Industrial Equipment, Department of Engineering Mechanics, Dalian University of Technology, Dalian 116024, China

*^b^* Department of Anatomy, College of Basic Medical Sciences, Dalian Medical University, Dalian 116044, China

**Quantified biochemical assay**

**Tab. S1 The collagen and GAG content of cartilage in different regions.**

| Component | Sample | Femur | | |  | Tibia | | | |
| --- | --- | --- | --- | --- | --- | --- | --- | --- | --- |
|  |  | FMI | FLI | FPI |  | TMI-M | TLI-M | TMI | TLI |
| Collagen  (μg/mg) | 1 | 15.39 | 14.33 | 18.37 |  | 19.46 | 14.00 | 15.72 | 11.41 |
|  | 2 | 19.33 | 19.98 | 21.60 |  | 21.41 | 17.37 | 15.52 | 22.14 |
|  | 3 | 41.52 | 46.67 | 44.15 |  | 30.05 | 39.09 | 35.06 | 24.85 |
| GAG  (μg/mg) | 1 | 9.33 | 15.85 | 12.47 |  | 13.65 | 17.30 | 15.96 | 11.96 |
|  | 2 | 12.31 | 15.77 | 22.39 |  | 21.84 | 18.49 | 23.31 | 19.21 |
|  | 3 | 18.38 | 18.17 | 17.62 |  | 18.24 | 20.58 | 28.97 | 26.51 |

**Constitutive model fitting**

The hyperelastic constitutive fitting of three models, *i.e.* Fung, Gent and Ogden, are performed for the mean stress-strain curves at strain rate 3%/min, 30%/min and 300%/min, respectively. The fitting results are shown in Fig. S1-3.


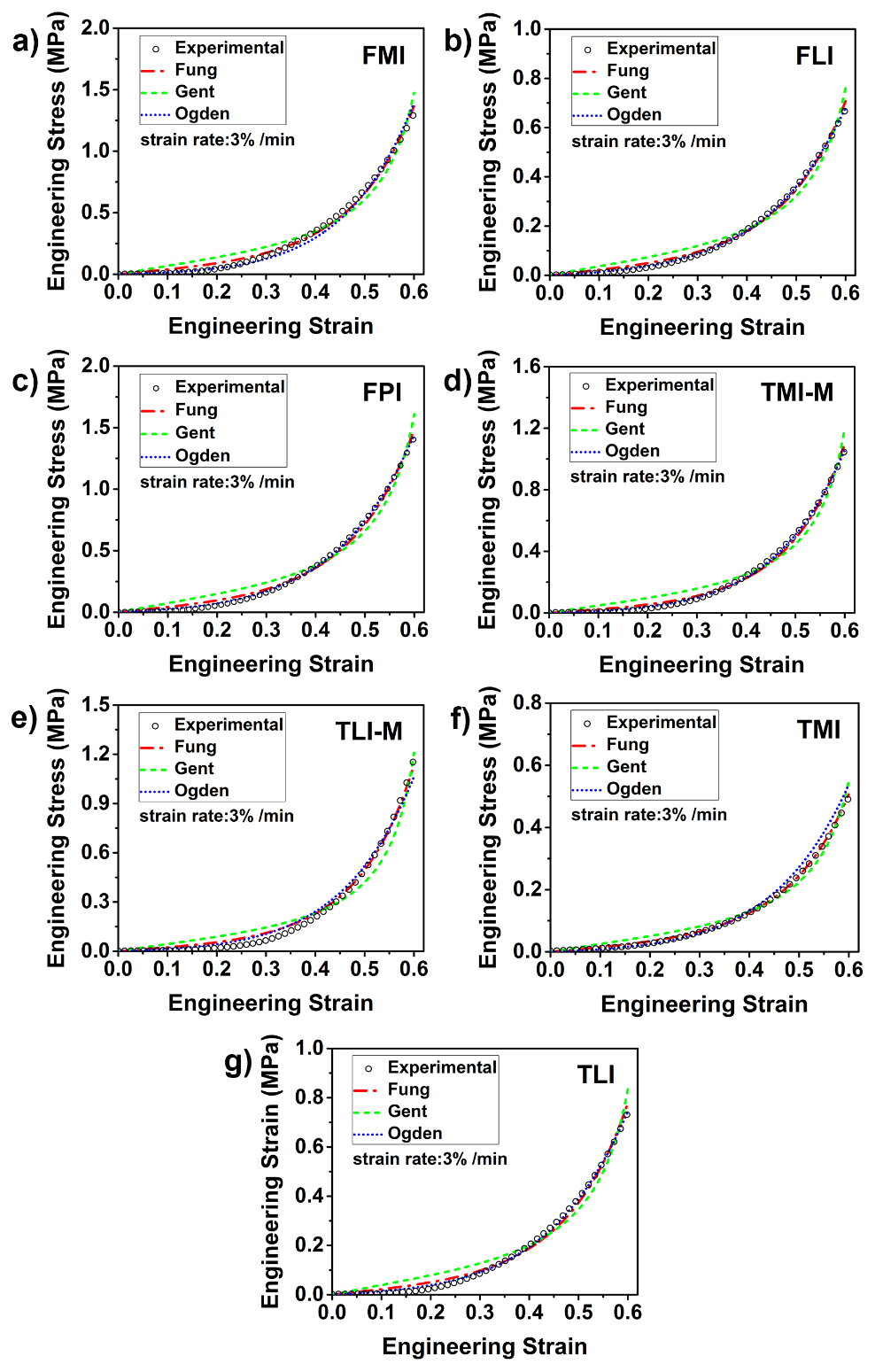


**Figure S1.** Plot of fit between three constitutive models and mean experimental data of predetermined regions of articular cartilage at strain rate 3%/min.


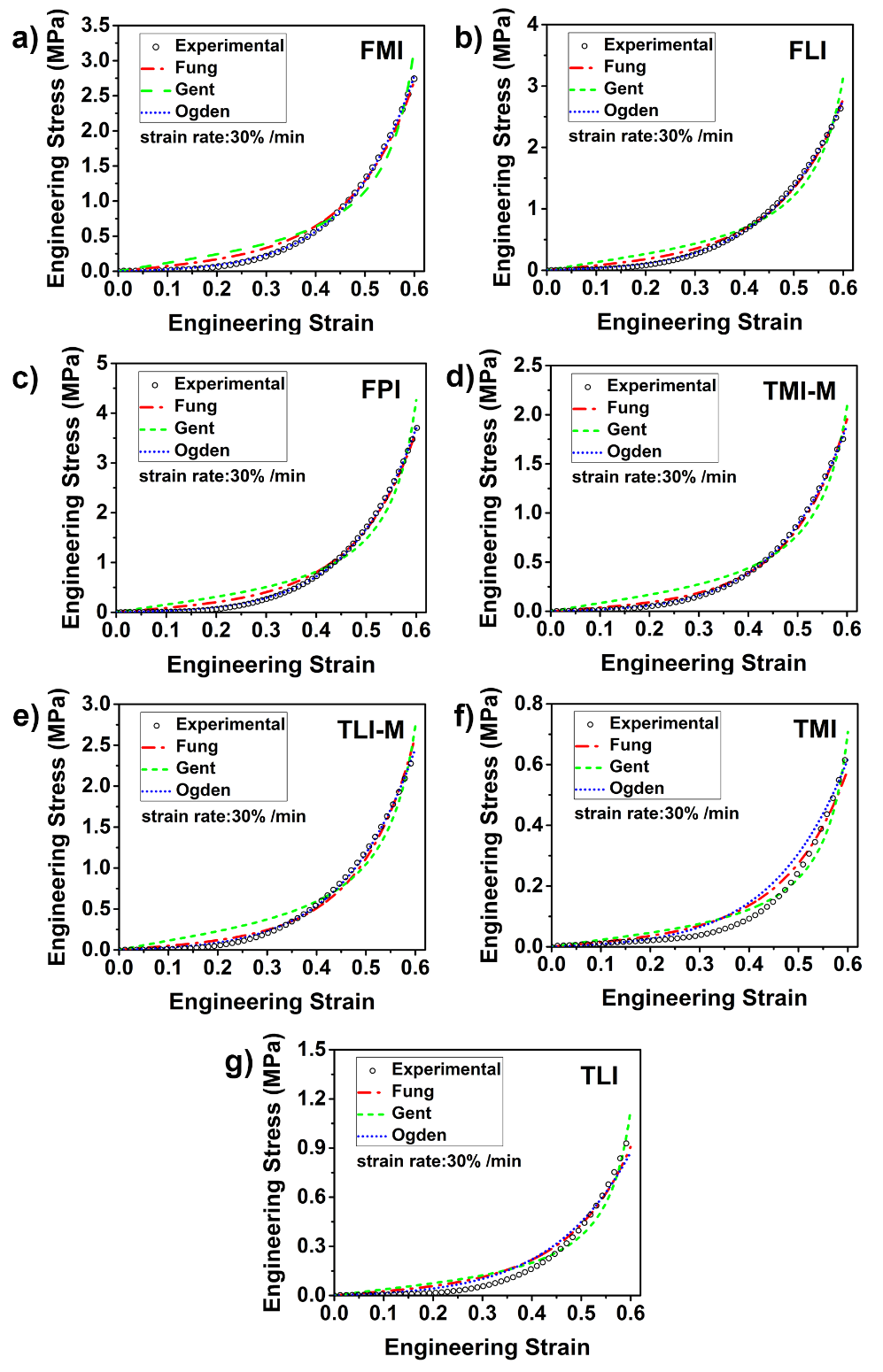


**Figure S2.** Plot of fit between three constitutive models and mean experimental data of predetermined regions of articular cartilage at strain rate 30%/min.


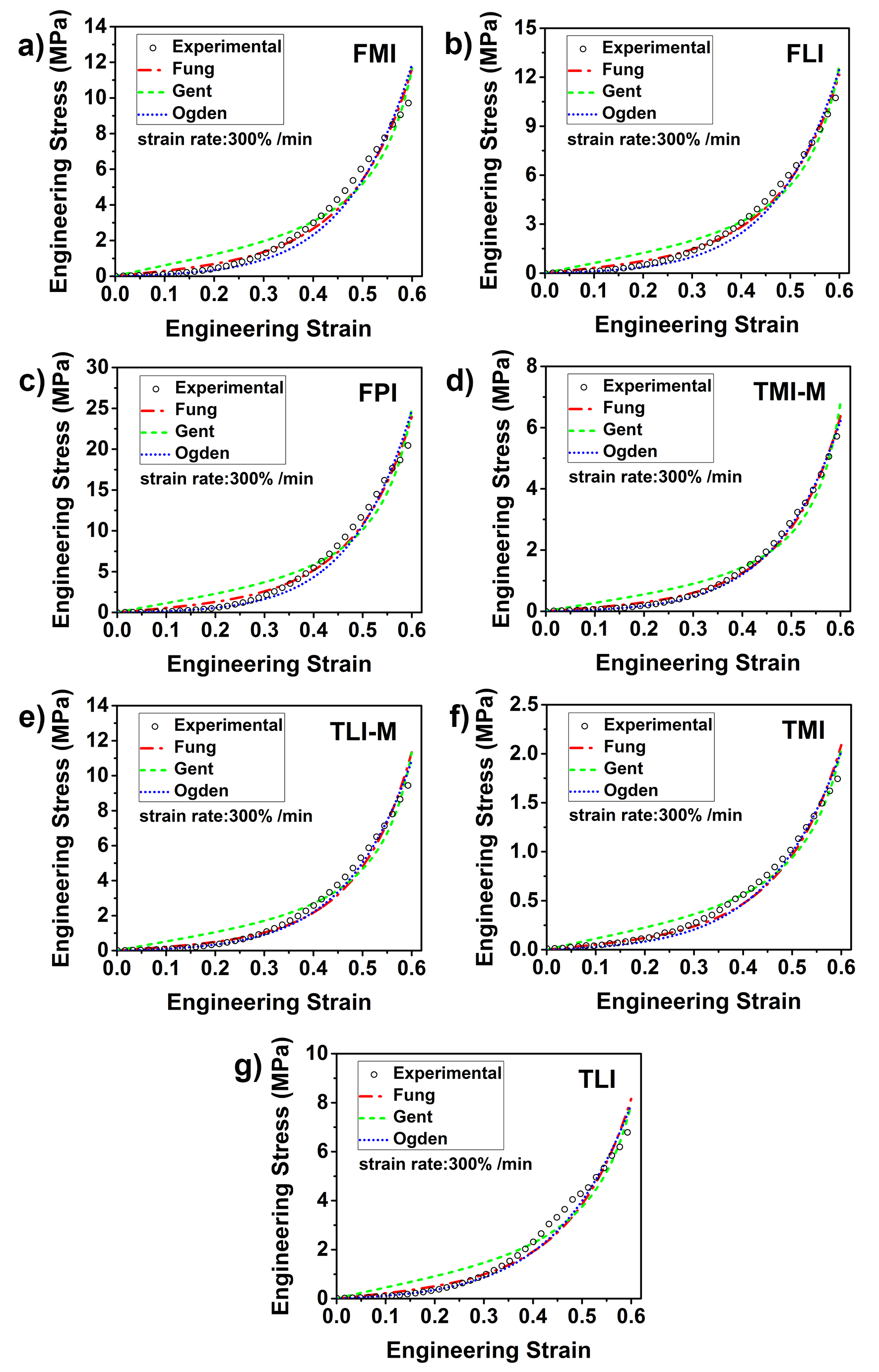


**Figure S3.** Plot of fit between three constitutive models and mean experimental data of predetermined regions of articular cartilage at strain rate 300%/min.

1. * Corresponding author. Tel: +86 411 84706353, E-mail：[wei.zhang@dlut.edu.cn](mailto:wei.zhang@dlut.edu.cn) [↑](#footnote-ref-1)
